# Optimal Timing of Targeted Temperature Management for Post-Cardiac Arrest Syndrome: Is Sooner Better?

**DOI:** 10.3390/jcm12072628

**Published:** 2023-03-31

**Authors:** I-Ting Wang, Chieh-Jen Wang, Chao-Hsien Chen, Sheng-Hsiung Yang, Chun-Yen Chen, Yen-Chun Huang, Chang-Yi Lin, Chien-Liang Wu

**Affiliations:** 1 Division of Pulmonary and Critical Care Medicine, Department of Internal Medicine, MacKay Memorial Hospital, Taipei 104217, Taiwan; 2 Department of Critical Care Medicine, MacKay Memorial Hospital, Taipei 104217, Taiwan; 3 Department of Medicine, MacKay Memorial College, New Taipei City 25245, Taiwan; 4 Ph.D. Program in Translational Medicine, National Taiwan University and Academia Sinica, Taipei 11529, Taiwan; 5 Division of Cardiology, Department of Internal Medicine, MacKay Memorial Hospital, Taipei 104217, Taiwan; 6 Graduate Institute of Business Administration, Fu Jen Catholic University, New Taipei City 242062, Taiwan; 7 Artificial Intelligence Development Center, Fu Jen Catholic University, New Taipei City 242062, Taiwan

**Keywords:** in-hospital cardiac arrest, out-of-hospital cardiac arrest, postcardiac arrest syndrome, targeted temperature management, timing

## Abstract

Targeted temperature management (TTM) is often considered to improve post-cardiac arrest patients’ outcomes. However, the optimal timing to initiate cooling remained uncertain. This retrospective analysis enrolled all non-traumatic post-cardiac arrest adult patients with either out-of-hospital cardiac arrest (OHCA) or in-hospital cardiac arrest (IHCA) who received TTM from July 2015 to July 2021 at our hospital. The values of time delay before TTM and time to target temperature were divided into three periods according to optimal cut-off values identified using receiver operating characteristic curve analysis. A total of 177 patients were enrolled. A shorter time delay before TTM (pre-induction time) was associated with a lower survival chance at 28 days (32.00% vs. 54.00%, *p* = 0.0279). Patients with a longer cooling induction time (>440 minis) had better neurological outcomes (1.58% vs. 1.05%; *p* = 0.001) and survival at 28 days (58.06% vs. 29.25%; *p* = 0.006). After COX regression analysis, the influence of pre-induction time on survival became insignificant, but patients who cooled slowest still had a better chance of survival at 28 days. In conclusion, a shorter delay before TTM was not associated with better clinical outcomes. However, patients who took longer to reach the target temperature had better hospital survival and neurological outcomes than those who were cooled more rapidly. A further prospective study was warranted to evaluate the appropriate time window of TTM.

## 1. Introduction

Targeted temperature management (TTM) has been the standard approach to improve postcardiac arrest outcomes for years [1,2]. However, several issues have yet to be clarified, including the optimal time to initiate cooling and the speed of reaching the target temperature. Almost all animal studies suggest that early interventions can improve survival and neurological outcomes [3]. However, human studies have shown that “the sooner the better” is not always the correct approach, especially if TTM is initiated during the arrest [4,5]. In addition, whether a shorter time delay between the return of spontaneous circulation (ROSC) and initiation of TTM is preferable also remains controversial, although most studies suggest that a shorter delay may be better [6,7,8,9]. In addition, some studies have reported that a faster cooling rate during TTM may be beneficial [7,8,9,10] for postcardiac arrest patients because of theoretical reasons [11] and variable cooling device efficiency [12]. However, other studies [13,14,15] have reported opposite results. Furthermore, some studies have shown that maintenance of afebrile instead of aggressive cooling does not lead to worse outcomes in these patients [16,17]. Our institution has adopted TTM for a number of years and previously reported the outcomes of TTM for out-of-hospital cardiac arrest (OHCA) [18] and in-hospital cardiac arrest (IHCA) [19] patients. The aim of this study was to analyze whether the timing and rate of TTM would modify the outcome of these patient groups.

## 2. Materials and Methods

### 2.1. Study Design

In this retrospective analysis, we enrolled all nontraumatic adult ROSC patients with either OHCA or IHCA who received TTM at Mackay Memorial Hospital (MMH) Taipei and Tamsui branches, Taiwan, from July 2015 to July 2021. The exclusion criteria were as follows: (1) those aged < 18 years; (2) those who died within 24 h after ROSC; (3) those who suffered from traumatic cardiac arrest; and (4) those who didn’t receive TTM. The study plan was reviewed and approved by the Institutional Review Board of MMH (approval reference No. 21MMHIS012e). The requirement for informed consent was waived because of the retrospective nature of the study.

### 2.2. Protocol Description

At MMH, every comatose ROSC patient admitted to the intensive care unit is considered a potential candidate for TTM. Permission for TTM is obtained from the patient’s next of kin. TTM was performed in accordance with the standard protocol at MMH (Supplement S1). Briefly, TTM was initiated with 4 °C Ringer’s lactate solution (30 mL/kg) given through an intravenous route, followed by wrapping the patient’s body in a surface cooling blanket (Arctic Sun Model 2000/5000, Medivance, Louisville, CO, USA) for 24 or 48 h. The targeted temperature is set at 33 °C. After the preset hypothermia phase was completed, the patients received controlled rewarming at a rate of 0.15 °C/h until 36.5 °C was reached. Active temperature management with medications to prevent fever (>37.2 °C) is allowed for another 24 h. Magnesium sulfate is routinely given to prevent shivering during the hypothermia phase. Propofol, fentanyl, and cisatracurium can be given according to the preference of the physician in charge.

### 2.3. Data Collection and Outcome Assessment

**Figure 1:**
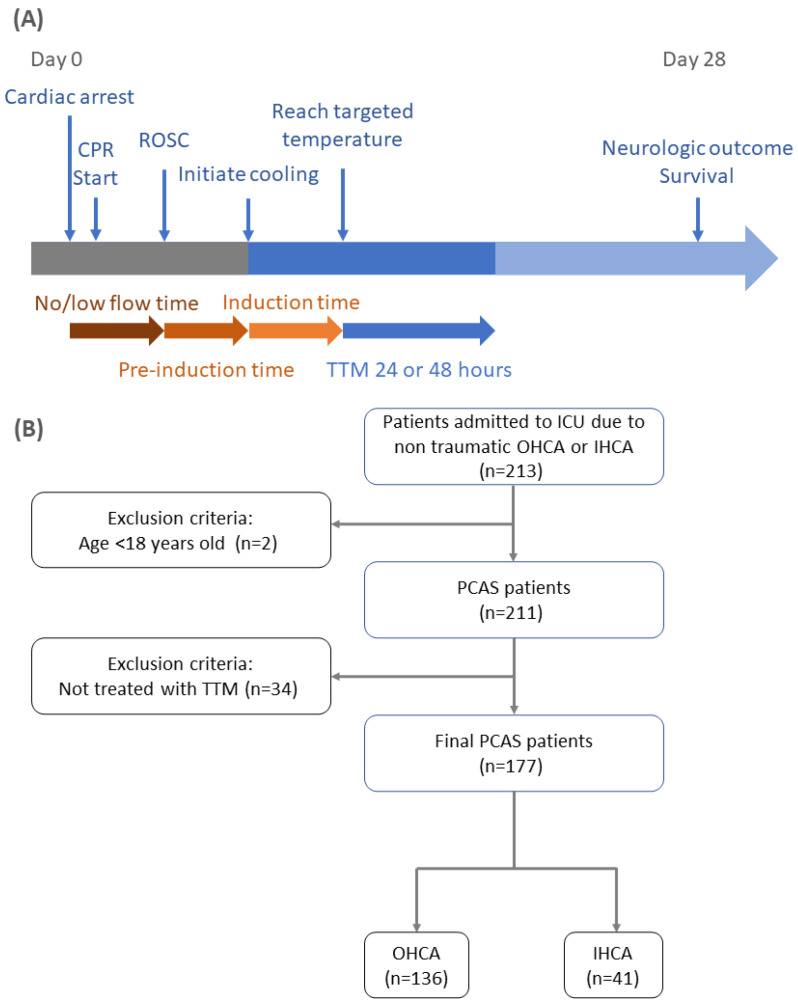
(**A**) Illustration of the time course from cardiac arrest to TTM. (**B**) Flowchart of patient inclusion and exclusion in the study. Abbreviations: CPR: cardiopulmonary resuscitation; ICU: intensive care unit; IHCA: in-hospital cardiac arrest; OHCA: out-of-hospital cardiac arrest; PCAS: post–cardiac arrest syndrome; ROSC: return of spontaneous circulation; TTM: targeted temperature management.

Data includes the patient’s age, sex, arrest type (IHCA or OHCA), whether or not the arrest was witnessed, presence of bystander chest compression, rhythm type when the emergency medical service staff arrived, epinephrine administration before ROSC, defibrillation, first available body temperature during cardiac arrest, and body temperature at the time of initiating cooling and end of cooling. The timing of the entire TTM course was recorded, including no-flow time (the time from cardiac arrest to initiation of cardiopulmonary resuscitation), low-flow time (the time from CPR to ROSC), pre-induction time (time from ROSC to initiation of cooling), and induction time (time from initiation of cooling to the achievement of the target temperature) (Figure 1A).

Two clinical outcomes were used in this study, namely, 28-day survival and neurological outcomes. Associations of induction time and rate of cooling with the two clinical outcomes were the secondary outcomes. Neurological outcomes were evaluated using the Cerebral Performance Category (CPC) scale as follows: category 1, good cerebral performance; category 2, moderate cerebral disability; category 3, severe cerebral disability; category 4, coma or vegetative state; and category 5, death/brain death. A CPC score of 3–5 was defined as a poor neurological outcome [20,21].

### 2.4. Statistical Analysis

Differences in baseline demographics between the survivors and non-survivors were analyzed using Pearson’s chi-squared test for categorical variables (presented as *n* (%)) and the *t*-test for continuous variables (presented as the mean ± SD). The Mann–Whitney U test was used for nonnormally distributed variables (presented as the median (interquartile range)). The pre-induction time and induction time were divided into three periods according to two optimal cut-off values identified using receiver operating characteristic (ROC) curve analysis [22,23,24]. The rate of cooling was calculated by dividing the difference between the temperature at ROSC and 33 °C by the time interval. The log-rank test was used for time-to-event analysis to compare differences in the survival probability between three groups of pre-induction and induction time. We also performed univariant and multivariant Cox regression models. All variables with *p* < 0.05 in the univariate Cox regression model were considered confounders and were included in the multivariant Cox regression model. All statistical tests were two-tailed, and *p*-values < 0.05 were considered statistically significant. Data extraction and processing were performed using SAS version 9.4 (SAS Institute Inc., Cary, NC, USA).

## 3. Results

### Population, Demographics, Arrest Characteristics

There are 213 nontraumatic cardiac arrest patients admitted to the intensive care unit and assessed. There are two patients excluded due to being less than 18 years old and 34 patients excluded from TTM treatment based on the criteria of our protocol. Finally, 177 PACS patients were enrolled, of whom 136 (76.8%) had OHCA and 41 (23.2%) had IHCA. (Figure 1B). Overall, 68 (38.42%) patients survived, and 109 (61.58%) patients died. There were no significant differences in 28-day survival (Supplement S2A–C) or neurological outcomes between the IHCA and OHCA groups (Supplement S3A–C). Besides, 116 patients (65.53%) underwent TTM for 24 h, and 61 patients (36.47%) underwent TTM for 48 h. Survival at 28 days was not significantly different in the 24-h group than in the 48-h group (57.76% vs. 54.10%, *p* = 0.8878).

The survivors had a significantly lower Acute Physiology and Chronic Health Evaluation (APACHE II) score (30.13 ± 6.29 vs. 32.62 ± 7.07, *p* = 0.0189), shorter no/low flow time (23.32 ± 20.71 vs. 39.97 ± 54.04 min, *p* = 0.0048), a slower rate of cooling (0.33 ± 0.18 vs. 0.42 ± 0.28°C/h, *p* = 0.0097) and longer time from cardiac arrest to target temperature (728.5 ± 363.85 vs. 614.79 ± 296.65 min, *p* = 0.0254) (Table 1). Twenty-five (15.52%) patients had good neurological outcomes, while 136 (84.47%) patients had poor neurological outcomes. The patients with good neurological outcomes were significantly younger (57.96 ± 13.59 vs. 66.02 ± 14.3 years. *p* = 0.0068), lower APACHE II score (27.4 ± 5.85 vs. 32.2 ± 6.66, *p* = 0.0007), shorter no/low flow time (20.52 ± 21.39 vs. 37.18 ± 49.47 min, *p* = 0.0107), longer induction time (350 ± 224.25 vs. 259.79 ± 223.95 min, *p* = 0.0225), higher body temperature at ROSC (36.34 ± 0.98 vs. 35.79 ± 1.34 °C, *p* = 0.0499), and higher body temperature at the start of cooling (36.68 ± 1.15 vs. 35.65 ± 1.56 °C, *p* = 0.0023) compared to the patients with poor neurological outcomes (Table 2).

**Figure 2:**
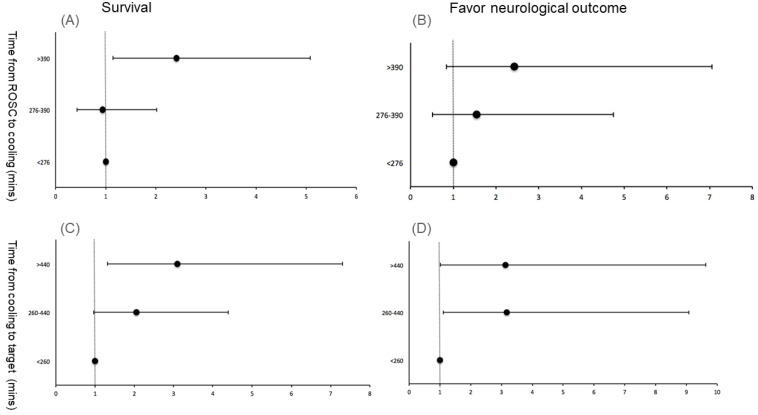
Adjusted odds ratio plot for survival and good neurological outcomes. (**A**) Time from ROSC to cooling for survival. (**B**) Time from ROSC to cooling for better neurological outcomes. (**C**) Time from cooling to target for survival. (**D**) Time from cooling to target for better neurological outcomes. Each black dots represent the odds ratio, and the horizontal line indicates the 95% confidence interval. Abbreviations: ROSC, return of spontaneous circulation.

ROC curve analysis was used to identify optimal cut-off values for pre-induction time, which we then divided into three groups: 0–276 min, 276–390 min, and >390 min (Supplement S4A). The rate of cooling was slower (0.50, 0.36, and 0.25°C/h in the 0–276, 276–390, and >390 min groups, respectively, *p* < 0.001), survival was better (32.00%, 32.69%, and 54.00%, *p* = 0.0279), and the mean CPC score was lower (4.40, 4.15, and 3.72, *p* = 0.0182) in the longer pre-induction group (Table 3 A). However, after multivariate logistic regression analysis, a pre-induction time > 390 min was associated with increased survival compared to <276 min (adjusted odds ratio [AOR]: 2.41, 95% confidence interval [CI]: 1.14–5.08, *p* = 0.02) (Table 3 B, Figure 2A,B).

Similarly, we used ROC curve analysis to identify the optimal cut-off values for induction time, which we also divided into three groups: 0–260 min, 260–440 min, and >440 min (Supplement S4B). The age was younger (68.1 ± 14.7, 62.9 ± 13.4, and 58.9 ± 16.2 years in the 0–260, 260–440, and >440 min groups, respectively, *p* = 0.0030), the rate of cooling was slower (0.48, 0.30, and 0.19 °C/h, respectively, *p* < 0.001), survival was better (29.25%, 47.50%, and 58.06%, respectively, *p* = 0.006), and the mean CPC score was lower (4.43, 3.85, and 3.48, respectively, *p* < 0.001), in the group of induction time > 440 min. The mean APACHE II score (33.19, 28.62 and 30.35, respectively, *p* = 0.0003) was lower in the 260–440 min induction time group (Table 4A). After multivariate logistic regression analysis, an induction time of >440 min was associated with increased survival compared to the <260 min group (AOR: 3.10, 95% CI: 1.32–7.30, *p* = 0.01). In addition, induction times of 260–440 and >440 min were associated with higher rates of good neurological outcomes than induction times < 260 min (AOR: 3.17, 95% CI: 1.11–9.08, *p* = 0.03, and AOR: 3.10, 95% CI: 1.02–9.63, *p* < 0.05, respectively) (Table 4B, Figure 2C,D).

**Figure 3:**
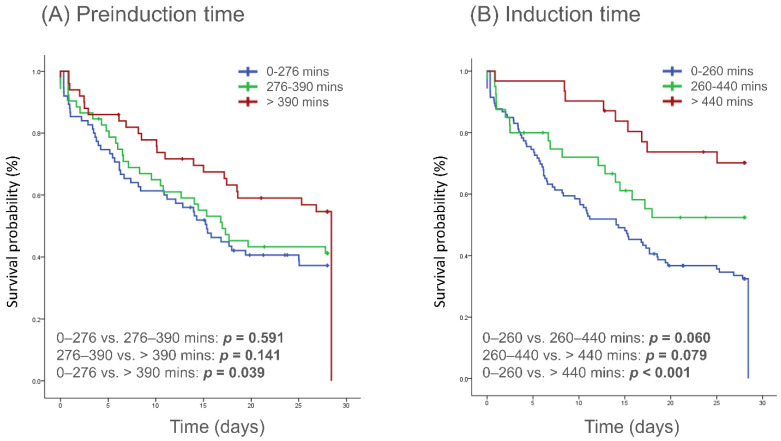
Kaplan–Meier curves of the cumulative probability of survival to day 28 after cardiac arrest according to different pre-induction (**A**) and induction time (**B**).

In Kaplan–Meier analysis, the group with pre-induction time > 390 min had significantly longer survival time compared to the group of 0–276 min in 28 days (*p* = 0.039) (Figure 3A), while marginal longer survival time in 90 days and 180 days. (Supplement S5A,C). The group with induction time > 440 min had significantly longer survival than the group of 0–260 min in 28 days (*p* < 0.001) (Figure 3B), 90 days (*p* = 0.001), and 180 (*p* = 0.001) days (Supplement S5B,D). In Cox univariant regression model, pre-induction time, induction time, age, initial rhythm, and APACHE II were significant and were considered confounders. After multivariate analysis, only induction time > 440 min had a significantly lower hazard ratio (0.382, 95% CI: 0.187–0.776, *p* = 0.008) to mortality through 28 days (Table 5).

## 4. Discussion

In this retrospective study of postcardiac arrest patients, we found that a faster drop in body temperature during TTM was associated with worse outcomes, whereas a shorter resuscitation time, slower cooling rate and lower APACHE II score at admission were associated with better hospital survival. In contrast to previous reports, we found that a shorter time delay before TTM and a faster rate of achieving the target temperature after TTM did not lead to better clinical outcomes. Moreover, we found that a slower initiation of TTM and a slower rate of reaching the temperature goal were associated with better outcomes, at least until the 28th day after cardiac arrest.

Timing is a critical part of resuscitation, and delays in treatment, including patient delay and prehospital system delay, have been shown to be major determinants of the outcomes of ST-elevation myocardial infarction patients [25]. Reducing the time delay to primary percutaneous coronary interventions among patients with ST-elevation myocardial infarction, even by <60 to 90 min, has been shown to significantly improve outcomes [26]. TTM has been widely used for postcardiac arrest patients to improve hospital survival and neurological outcomes in the last decade [1,2]. However, the optimal timing remains uncertain. The mechanisms of hypothermia can mitigate brain injury at multiple levels, including the ability to reduce cerebral metabolism by 6–10% for each 1 °C reduction in body temperature [11,27]. This is thought to be the main protective effect since oxygen deprivation, and the accumulation of excitatory neurotransmitters play a significant role in cell death after cerebral ischemia. Another important effect is the suppression of inflammatory responses and associated free radical production during ischemia and repercussion injury [11]. Some experimental studies have suggested that hypothermia can mitigate the activation of apoptosis [28,29]. White et al. reported increased levels of arterial neuroprotection D1 in animal cardiac arrest models after 3 h of resuscitation, [30] and the level was three times higher in hypothermic animals. Accordingly, the early implementation of TTM after cardiac arrest, and even during arrest, has been shown to be beneficial, at least in animal studies [9,31]. Sendelbach et al. reported that the odds of a poor neurological outcome increased with each 5-min delay in initiating TTM (OR = 1.06, 95% CI 1.02–1.10) [9]. In a post hoc analysis of nontraumatic OHCA patients, Stanger et al. found better neurological outcomes in the early door-to-TTM group (<122 min, 48% and 38%, respectively) [32]. However, early TTM in human trials has not always shown positive results. The PRINCESS trial reported that intra-arrest cooling initiated <20 min from the collapse was not associated with better neurological outcomes compared to cooling initiated at the hospital [33]. Moreover, Bernard et al. found that patients with prehospital cold fluid infusion tended to have worse outcomes [6]. In the present study, we found that the delayed initiation of TTM was associated with a higher short-term survival rate than a shorter pre-induction time, but there were no significant differences in good/poor neurological outcomes between the groups. Further studies are warranted to investigate these conflicting results.

Some animal studies have suggested that a pre-induction delay did not significantly influence the outcomes of TTM [34,35]. Apoptosis can develop later during the postperfusion phase and continue for at least 48 h, [29,36] which suggests a wide window of opportunity to mitigate this pathway. Lawrence et al. reported that the neuroprotective effect of hypothermia is both optimal and equivalent when initiated between 1 and 8 h after reoxygenation [37]. Che et al. also reported that a delay of up to 4 h did not significantly influence outcomes [38]. In a human study, Perman et al. reported no significant difference in pre-induction time between patients with good versus poor outcomes [14]. Our results showed that patients with a shorter pre-induction delay (<276 min) had a nearly two-fold higher risk of death than those with a delay > 390 min. This suggests that while hypothermia has a wide spectrum of pathophysiologic protective mechanisms, there may also be deleterious effects. Singh et al. reported that hypothermia might protect against conditions that generate reactive oxygen and nitrogen species and that this may decrease the activity of cellular antioxidant defenses to attenuate the benefits of TTM [39]. A previous observational study also suggested that if TTM was started within 2 h of cardiac arrest, the mortality rate was higher than if TTM was started later [40]. Since TTM may have limited benefits after 12 h due to irreversible damage to nerve cells, [41] there may be an optimal time frame to initiate TTM after ROSC. Our results suggest this possibility; however, further investigations are needed for verification.

Several studies have suggested that the clinical benefits of TTM in postcardiac arrest patients depended on whether the temperature target was achieved rapidly [7,8,9,10]. However, other studies have reported opposite results. In a retrospective study, Haugk et al. found that cooling to 34 °C or below was slower (median time: 209 min) in patients with favorable neurological outcomes than in those with unfavorable outcomes (158 min) [13]. In addition, Perman et al. reported that patients with a longer induction time (>300 min to reach 33 °C) were associated with better neurological outcomes than those with an induction time of <120 min [14]. Lin et al. also observed that a slower rate of cooling was associated with improved neurological outcomes (OR, 0.73 °C/h) and survival [15]. Our study results are consistent with these findings, and patients with a longer induction time, especially >440 min, had a better survival rate (AOR, 3.10) than those with an induction time < 260 min on the 28th day after cardiac arrest. The survival benefit of this group of patients remained significant until 180 days after cardiac arrest. Moreover, they were more likely to have good neurological outcomes compared with those who were cooled rapidly. The cooling rate was 0.2 °C/h in this group, almost half that of the rate in the <260 min group (0.48 °C/h). One possible explanation may be compromised thermoregulation after cerebral insult [13,14,42,43]. Among our patients, those who were cooled more rapidly had significantly higher APACHE II scores than those who were cooled more slowly. These characteristics are consistent with the aforementioned hypothesis. Besides, Benz-Woerner et al. found that patients with poor neurological outcomes had a lower core temperature upon ROSC [44]. Our results are in line with those of Lin et al. [15], and the patients with good neurological outcomes had a higher core temperature upon ROSC. An intact thermoregulatory center allows the body to exhibit a normal response to temperature differences. This may explain why shivering [45] and bradycardia [46] are more common in patients with improved neurological outcomes during TTM. In summary, our patients with good neurological outcomes were younger, had a lower APACHE II score, higher temperature upon ROSC, and shorter no/low flow time, which are in line with the findings of Perman et al. [14]. Thus, we hypothesize that a TTM target at <34 °C might be a ‘stress test’ to recognize the integrity of the patient’s thermoregulatory center and that the cerebral insult severity after cardiac arrest itself is the main prognostic factor. This speculation was in line with the finding of the TTM-2 trial [17]. In the sub-group analysis of the TTM-2 trial, [47] Simpson et al. divided included sites into six groups according to the speed of hypothermia, which was surrogated as the average temperature at four hours from ROSC in the hypothermia group. Of the patients enrolled in the fastest sites, whose average temperature was ≤34 °C at four hours, 49% of the patients died at six months in the hypothermia group compared with 46% in the normothermia group. Compared to the patients enrolled in the slowest sites, whose average temperature was 35.3, the mortality rate was 45% in the hypothermia group and 56% in the normothermia group. The patients with faster speed to target temperature didn’t show better survival compared to those with slower speed. Our study also found that the non-survivors had a faster rate of cooling compared to survivors (0.42 ± 0.28 vs. 0.33 ± 0.18 °C/h, *p* = 0.0097). However, there are inherent limitations because both studies were post-hoc analyses or retrospective, and the number may be too small to be underpowered. Further investigations are warranted to investigate whether a lower temperature target in TTM is necessary for postcardiac arrest patients.

This study has some limitations. First, it is a retrospective study, and thus missing data are inevitable. It was especially difficult to estimate no/low flow time as we did not have access to the emergency medical service database. Second, the average pre-induction time delay in our patient groups was approximately 6 h, even in the IHCA group. The most common causes of delay were emergency room—hospital ward—ICU preparation for patient transfer, surrogate availability to make decisions, and overcrowding. However, our institution has long-term experience in TTM and has used the same protocol for a decade [18,19]. The performance was not inferior to other similar studies of the same period. We were surprised to find that delays in pre-induction and induction times of TTM may be associated with better survival, albeit short-term. However, most experts believe that “the sooner the better”, [41,48,49,50] the temperature target, devices used for TTM, patient arrest location, no/low flow time limits and exclusion criteria have varied between studies [1,2,4,6,7,8,9,10,11,14,15,18,19,22,32,33,40,51,52]. However, the length of the delay of pre-induction time becomes statistically insignificant after multiple Cox regression analysis. There might be a ‘time window’ in the timeline of patient management, which suggests that an adequate delay may be able to attenuate the side effects of early cooling without compromising the protective effects of hypothermia. But too much delay can be detrimental again. An international registration network to record timing issues with TTM would help to elucidate this issue. Third, 61 (36.5%) of our patients received 48 h instead of 24 h TTM which may interfere with clinical outcomes [53,54,55]. However, our results were consistent with the results of the latest randomized clinical trial; [56] target temperature management for 48 h did not significantly improve clinical outcomes compared with those received TTM for 24 h.

## 5. Conclusions

Among the enrolled patients with OHCA/IHCA who received TTM in this retrospective study with a limited sample size, we found that a shorter pre-induction delay was not associated with improved survival outcomes. Patient outcomes were primarily determined by disease severity and no/low flow time. The patients with a longer time delay before TTM initiation had a higher hospital survival rate, but their neurological outcomes were not different from those with a shorter delay. Patients with a longer induction time had a higher hospital survival rate and better neurological outcomes. However, our results were not suggesting clinicians delay or abandon TTM, and further prospective study was warranted to evaluate the appropriate time window of TTM.

## Figures and Tables

**Table 1:** Comparisons of the survivors and non-survivors at day 28 with IHCA/OHCA.

	Total *n* (%)	Non-Survivors (*n* = 109)		Survivors (*n* = 68)		*p*
		OHCA (*n* = 76)	IHCA (*n* = 33)	OHCA (*n* = 60)	IHCA (*n* = 8)	
Age (mean, std), years	65.55 (15.10)	67.25 (15.03)		62.83 (14.09)		0.0581
Sex, Male (number, percentage)	109 (61.58)	68 (62.39)		41 (60.29)		0.7807
APACHE II (mean, std)	31.67 (6.70)	32.62 (7.07)		30.13 (6.29)		0.0189
Rate of cooling, °C/h (mean, std)	0.39 (0.25)	0.42 (0.28)		0.33 (0.18)		0.0097
BT at cardiac arrest, min (mean, std)	36.23 (1.20)	36.25 (1.20)		36.19 (1.21)		0.7495
BT at ROSC, min (mean, std)	35.87 (1.31)	35.72 (1.39)		36.1 (1.13)		0.0674
BT at start of cooling, min (mean, std)	35.79 (1.55)	35.61 (1.62)		36.07 (1.40)		0.0539
From CPR to ROSC, min (no/low flow time) (mean, std)	33.46 (44.77)	39.97 (54.04)		23.32 (20.71)		0.0048
From ROSC to initial cooling, min (pre-induction time) (mean, std)	350.27 (216.72)	322.64 (174.08)		394.5 (267.0)		0.0510
From cooling to target, min (induction time) (mean, std)	270.90 (229.53)	246.13 (229.27)		310.6 (225.9)		0.0690
Arrest to TTM target, min (mean, std)	659.22 (328.29)	614.79 (296.65)		728.5 (363.85)		0.0254

Abbreviations: OHCA: out-of-hospital cardiac arrest; IHCA: in-hospital cardiac arrest; APACHE II: acute physiology and chronic health evaluation II; BT: body temperature; ROSC: return of spontaneous circulation; CPR: cardiopulmonary resuscitation; TTM: targeted temperature management.

**Table 2:** Comparisons of the patients with IHCA/OHCA with good and poor neurological outcomes at day 28.

	Good Neurological Function (*n* = 25)		Poor Neurological Function (*n* = 136)		*p*
	OHCA (*n* = 21)	IHCA (*n* = 4)	OHCA (*n* = 102)	IHCA (*n* = 34)	
Age, years, (mean, std)	57.96 (13.59)		66.02 (14.3)		0.0068
Sex, Male (number, percentage)	18 (72)		85 (62.5)		0.4948
APACHE II (mean, std)	27.4 (5.85)		32.2 (6.66)		0.0007
Rate of cooling, °C/h (mean, std)	0.015 (0.012)		0.0266 (0.036)		0.096
BT at cardiac arrest, min (mean, std)	36.47 (1.01)		36.26 (1.21)		0.6008
BT at ROSC, min (mean, std)	36.34 (0.98)		35.79 (1.34)		0.0499
BT at start of cooling, min (mean, std)	36.68 (1.15)		35.65 (1.56)		0.0023
From CPR to ROSC, min (no-low flow time) (mean, std)	20.52 (21.39)		37.18 (49.47)		0.0107
From ROSC to initial cooling (min) (pre-induction time)	401.84 (324.76)		346.85 (201.20)		0.239
From cooling to target, min (induction time) (mean, std)	350 (224.25)		259.79 (223.95)		0.0225
Arrest to TTM target, min (mean, std)	772.36 (403.70)		648.40 (319.11)		0.107

Abbreviations: OHCA: out-of-hospital cardiac arrest; IHCA: in-hospital cardiac arrest; APACHE II: acute physiology and chronic health evaluation II; BT: body temperature; ROSC: return of spontaneous circulation; CPR: cardiopulmonary resuscitation; TTM: targeted temperature management.

**Table 3:** (**A**) Comparisons of the three pre-induction time groups. (**B**) Adjusted odds ratios of different pre-induction times for survival and good neurological outcomes.

(A)				
	Pre-Induction Time			*p*
	0–276 Min (*n* = 75)	276–390 Min (*n* = 52)	>390 Min (*n* = 50)	
Age, years (mean, std)	67.5 (15.5)	63.2 (16.7)	65.2 (12.4)	0.2761
Gender, male (number, percentage)	48 (64.0)	31 (59.6)	30 (60.0)	0.8508
APACHE II score (mean, std)	32.13 (6.54)	32.83 (7.03)	30.77 (6.59)	0.2998
Rate of cooling (mean, std)	0.50 (0.31)	0.36 (0.16)	0.25 (0.13)	<0.001
CPC score 28 days after cardiac arrest (mean, std)	4.40 (1.10)	4.15 (1.41)	3.72 (1.44)	0.0182
28-day survival (mean, std)	24 (32.00)	17 (32.69)	27 (54.00)	0.0279
**(B)**				
Time from ROSC to Cooling * (min)	**Odds Ratio**	**95% CI**		***p* Value**
**Odds ratios for survival**				
<276	1	(Reference group)		
276–390	0.94	0.43–2.02		0.87
>390	2.41	1.14–5.08		0.02
**Odds ratios for good neurological outcomes**				
<276	1	(Reference group)		
276–390	1.55	0.51–4.75		0.44
>390	2.43	0.84–7.96		0.1

* Model adjusted for age, sex and time from ROSC to cooling. Abbreviation: APACHE: acute physiology and chronic health evaluation II; CI: confidence interval; CPC: cerebral performance category; ROSC, return of spontaneous circulation.

**Table 4:** (**A**) Comparisons of the three induction time groups. (**B**) Adjusted odds ratios of different induction times for survival and good neurological outcomes.

(A)				
	Induction Time			*p*
	0–260 Min (*n* = 106)	260–440 Min (*n* = 40)	>440 Min (*n* = 31)	
Age, years (mean, std)	68.1 (14.7)	62.9 (13.4)	58.9 (16.2)	0.0030
Gender, male (number, percentage)	62 (58.5)	24 (60.0)	23 (74.2)	0.2788
APACHE II score(mean, std)	33.19 (6.46)	28.62 (6.04)	30.35 (6.82)	0.0003
Rate of cooling (mean, std)	0.48 (0.28)	0.30 (0.12)	0.19 (0.10)	<0.001
CPC score 28 days after cardiac arrest (mean, std)	4.43 (1.05)	3.85 (1.52)	3.48 (1.58)	<0.001
28-day survival (mean, std)	31 (29.25)	19 (47.50)	18 (58.06)	0.0060
**(B)**				
**Time from Cooling to Target ^#^** **(min)**	**Odds Ratio**	**95% CI**		***p* Value**
**Odds ratio for survival**				
<260	1	(Reference group)		
260–440	2.05	0.96–4.39		0.06
>440	3.10	1.32–7.30		0.01
**Odds ratio for good neurological outcomes**				
<260	1	(Reference group)		
260–440	3.17	1.11–9.08		0.03
>440	3.10	1.02–9.63		<0.05

Abbreviation: APACHE II: Acute Physiology and Chronic Health Evaluation II; CI: confidence interval; CPC: cerebral performance category. ^#^ Model adjusted for age, sex, and time from cooling to target.

**Table 5:** Univariant and multivariant Cox model results for mortality at 28 days.

	Univariate		Multivariate	
	Hazard ratio (95% CI)	*p* Value	Odds Ratio (95% CI)	*p* Value
Pre-induction time				
0–276 min	1 (Reference group)		1 (Reference group)	
276–390 min	0.902 (0.572–1.423)	0.657	0.907 (0.570–1.444)	0.681
>390 min	0.594 (0.358–0.984)	0.043	0.636 (0.382–1.057)	0.081
Induction time				
0–260 min	1 (Reference group)		1 (Reference group)	
260–440 min	0.646 (0.389–1.072)	0.091	0.781 (0.458–1.332)	0.364
>440 min	0.304 (0.152–0.609)	0.001	0.382 (0.187–0.776)	0.008
Age	1.016 (1.002–1.030)	0.026	1.005 (0.990–1.021)	0.513
Sex, male	1.108 (0.741–1.655)	0.618		
Witnessed	0.738 (0.493–1.104)	0.618		
Initial rhythm, shockable	0.463 (0.267–0.802)	0.006	0.600 (0.336–1.071)	0.084
Basic life support	0.921 (0.539–1.574)	0.763		
Duration of resuscitation effort	1.002 (0.998–1.006)	0.248		
APACHE II	1.037 (1.007–1.067)	0.014	1.019 (0.986–1.053)	0.26

APACHE II: acute physiology and chronic health evaluation, CI: confidence interval.

## Data Availability

The data sets analyzed during the current study are available from the corresponding author upon reasonable request.

## Supplementary Materials

The following supporting information can be downloaded at: https://www.mdpi.com/article/10.3390/jcm12072628/s1, Supplement S1: Protocol of targeted temperature management in MacKay Memorial Hospital.; Supplement S2: 28-day survival in the IHCA and OHCA groups; Supplement S3: Neurological outcomes in the IHCA and OHCA groups; Supplement S4: ROC curve analysis for optimal cut-off values of pre-induction and induction time; Supplement S5: Kaplan–Meier curves of the cumulative probability of survival to day 90 and day 180 after cardiac arrest according to different pre-induction and induction time.
