# Supplementary material for: Optimal Timing of Targeted Temperature Management for Post-Cardiac Arrest Syndrome: Is Sooner Better?
Source: J Clin Med. 2023 Mar 31;12(7):2628. doi: 10.3390/jcm12072628 (PMC10095041; doi:10.3390/jcm12072628)

Supplement S1. Protocol of targeted temperature management in MacKay Memorial Hospital.

<Cooling Protocol ArcticSun > (Cooling for 24 hours or 48 hours)

Start on ____ yyyy ____mm _____dd AM / PM_____:_____

Any of the following contraindications?

[Yes / No]

a. Awake spontaneously

b. Pregnancy

c. Initial BT < 30°C

d. Terminal illness

e. Primary intracranial event

■■■■■■■■■■■■■■■■■■■■■■■■■■■■■■■■■■■■

Initial 5-R Score [Total score: ____]

□ (2) a. initial rhythm (VT/VF)

□ (1) b. starting Resuscitation (< 5 mins)

□ (2) c. Return of spontaneous circulation (< 30 mins)

□ (1) d. light Reflex

□ (1) e. absent of re-arrest

■■■■■■■■■■■■■■■■■■■■■■■■■■■■■■■■■■■■

rCAST Score [Total score: ____]

Categorization of each variable

Score	0	1	2	3
Initial rhythm	shockable	non-shockable		
Witness / until ROSC time	<20 mins	≥20 mins	No witness	
PH	≥7.31	7.30-7.16	7.15-7.01	≦7.00
Lactate	≦5.0	5.1-10.0	10.1-14.0	≥14.1
GCS M	≥2	1		

Formula for calculation of rCAST

1.0 x (initial rhythm score) + 2.0 x (witness/until ROSC time score) +2.5x (PH score)+0.5 (Lactate score) + 4.5x (GCS M score) = rCAST core points

■■■■■■■■■■■■■■■■■■■■■■■■■■■■■■■■■■■■

[Cooling phase]

Frozen L/R (4°C) ___ml IV status (30 ml/kg)

Insert anal or esophageal temperature probe

Check vital signs and record the shivering Q1H until BT < 33°C and then Q2H during the cooling phase.

On ArcticSun machine

1. Set target 33°C for 24 hrs 48 hrs

2. MgSO4 (10%, 20 ml) 5 amps in normal saline 240 cc, IVD 10 cc/hr for one day
3. Acetaminophen 1# Q4H for 48 hours
4. Q10 (10 mg) 3# Tid for 5 days
5. Check serum Na, K, Cl Q8H; Mg and Ca QD throughout the course

If the patient has shivering:
1. start Propofol (200 mg/20 ml, 10 mg/mL)→0.5-1 mg/kg loading dose →Initial 10 mcg/kg/min→Titrate by 5 mcg/kg/min Q 10 mins → Max dose 60 mcg/kg/min
3. If still non-control, then:
Stat IV Cisatracrium (10 mg/5 mL) → 0.1 mg/kg IV bolus→ 3 mcg/kg/min initially→ Titrate by 1 mcg/kg/min→ Max dose 10 mcg/kg/min

■■■■■■■■■■■■■■■■■■■■■■■■■■■■■■■■■■■

[Rewarm phase]____yyy ____mm___ dd AM / PM_____:_____

·After 24 hrs or 48 hrs from the beginning, rewarming was started, and rewarming was set at a rate of 0.15°C/hr, with a target of 36.5°C.

■■■■■■■■■■■■■■■■■■■■■■■■■■■■■■■■■■■

[End phase] _____yyyy ____mm____ dd AM / PM_____:_____

·Remove the ArcticSun machine 24 hr after beginning the rewarm

·Acetaminophen 650 mg via tube or rectum every 6 hours prn temperature > 37.2°C to control fever for the first 24 hours after re-warming is completed.

Supplement S2A. Non-survivor vs. survivor groups

	**Non-survivors** (N=109)	**Survivors** (N=68)	P
Age (mean, std)	67.25(15.03)	62.83(14.09)	0.0581
Gender, Male	68(62.39)	41(60.29)	0.7808
APACHE II (mean, std)	32.62(7.07)	30.31(6.29)	<0.001
Rate of cooling (°C/h)	0.42(0.28)	0.33(0.18)	0.0097
BT at cardiac arrest (min)	36.25(1.20)	36.19(1.21)	0.7495
BT at ROSC (min)	35.72(1.39)	36.1(1.13)	0.0674
BT at cool start (min)	35.61(1.62)	36.07(1.40)	0.0539
No/low flow time (min)	33.47(42.38)	21.48(16.29)	0.0096
Pre-induction time (min)	330.60(173.1)	396.39(266.20)	0.0727
Induction time(min)	246.13(229.27)	310.6(225.9)	0.0690
Arrest to TTM target (min) (mean, std)	614.79(296.65)	728.5(363.85)	0.0254

Supplement S2B. OHCA vs. IHCA in the non-survivor group

	Non-survivors (N=109)		P
	**OHCA** (n=76)	**IHCA** (n=33)	
Age (mean, std)	66.84(15.91)	68.12(12.95)	0.6642
Gender, Male	46(60.53)	22(66.67)	0.5432
APACHE II (mean, std)	29.87(7.29)	32.13 (5.03)	0.285
Rate of cooling (°C/h)	0.36(0.21)	0.55(0.37)	0.0105
BT at cardiac arrest (min)	35.91(1.07)	37.03(1.13)	<0.001
BT at ROSC (min)	35.46(1.29)	36.33(1.45)	0.0023
BT at cool start (min)	35.41(1.61)	36.07(1.57)	0.0514
No/low flow time (min)	34.58(29.41)	31.00(62.75)	0.7557
Pre-induction time (min)	331.20(173.20)	329.20(175.10)	0.9555
Induction time(min)	245.60(226.10)	247.50(240.10)	0.9680
Arrest to TTM target (min) (mean, std)	622.93(298.83) (n=73)	596.78(295.53)	0.6765

Supplement S2C. OHCA vs. IHCA in the survivor group

	Survivors (N=68)		P
	**OHCA** (n=60)	**IHCA** (n=8)	
Age (mean, std)	63.48(14.68)	58(16.75)	0.3322
Gender, Male	35(58.33)	6(75)	0.4630
APACHE II (mean, std)	32.49(5.78)	32.94(7.44)	0.757
Rate of cooling (°C/h)	0.33(0.18)	0.33(0.19)	0.9840
BT at cardiac arrest (min)	36.05(1.15)	37.26(1.20)	0.0075
BT at ROSC (min)	35.97(1.13)	37.01(0.75)	0.0147
BT at cool start (min)	36.07(1.39) (n=59)	36.13(1.58)	0.9014
No/low flow time (min)	21.96(14.77)	17.87(26.15)	0.6768
Pre-induction time (min)	380.30(257.60)	516.90(316.80)	0.1748
Induction time(min)	296.90(196.40)	413.60(387.0)	0.4275
Arrest to TTM target (min) (mean, std)	699.20(338.70)	948.40(487.70)	0.1992

Abbreviations: OHCA: out-of-hospital cardiac arrest; IHCA: in-hospital cardiac arrest; APACHE II: acute physiology and chronic health evaluation II; BT: body temperature; ROSC: return of spontaneous circulation; CPR: cardiopulmonary resuscitation; TTM: targeted temperature management

Supplement S3A. Good vs. poor neurologic outcome groups

	**Good neurologic outcome** (N=25)	**Poor neurologic outcome** (N=136)	P
Age (mean, std)	57.96(13.59)	66.02 (14.3)	0.0068
Gender, Male	18(72)	85 (62.5)	0.4948
APCHE II (mean, std)	27.4(5.85)	32.2(6.66)	0.0007
Rate of cooling (°C/h)	0.015(0.012)	0.2666 (0.036)	0.096
BT at cardiac arrest	36.47(1.01)	36.26(1.21)	0.6008
BT at ROSC	36.34(0.98)	35.79(1.34)	0.0499
BT at cool start	36.68(1.15)	35.65(1.56)	0.0023
No-low flow time	20.52(21.39)	37.18 (49.47)	0.0107
Pre-induction time (mean, std)	401.84(324.76)	346.85(201.20)	0.239
Induction time	350(224.25)	257.79(223.95)	0.0225
Arrest to TTM target	772.36(403.70)	640.24(311.50)	0.107

Supplement S3B. OHCA vs. IHCA in the good neurologic outcome group

	Good neurologic outcome 1, 2 (N=25)		P
	**OHCA** (n=21)	**IHCA** (n=4)	
Age (mean, std)	58.38(12.51)	55.75(20.61)	0.7308
Gender, Male	15(71.43)	3(75)	0.5432
APCHE II (mean, std)	6.66(2.81)	9(3.16)	0.1489s
Rate of cooling (°C/h)	0.31(0.13)	0.45(0.21)	0.09
BT at cardiac arrest	36.28(0.88)	37.42(1.24)	0.0360
BT at ROSC	36.20(0.97)	37.12(0.69)	0.0856
BT at cool start	36.73(1.04) (n=20)	36.42(1.78)	0.6351
No-low flow time	17.00(13.88)	28.00(36.30)	0.5901
Pre-induction time (mean, std)	425.50(348.90)	288.50(90.44)	0.1375
Induction time	339.5(203.9)	405(346.8)	0.6032
Arrest to TTM target	782(420.8)	721.5(345.2)	0.7899

Supplement S3C. OHCA vs. IHCA in the poor neurologic outcome group.

	Poor neurologic outcome 3, 4, 5. (N=136)		P
	**OHCA** (n=102)	**IHCA** (n=34)	
Age (mean, std)	66.63(15.60)	67.35(13.17)	0.8015
Gender, Male	66(57.39)	25(67.57)	0.2720
APCHE II (mean, std)	10.38(3.57)	11.27(3.54)	0.1895
Rate of cooling (°C/h)	0.36(0.21)	0.52(0.37)	0.0168
BT at cardiac arrest	35.92(1.14)	37.04(1.13)	<0.001
BT at ROSC	35.59(1.26)	36.50(1.40)	0.0014
BT at cool start	35.52(1.55)	36.04(1.55)	0.0753
No-low flow time	35.94(40.61) (n=112)	28.48(59.62)	0.7952
Pre-induction time (mean, std)	339.60(179.60)	374.20(227.70)	0.3433
Induction time	255.2(214.4)	266.4(270.8)	0.8193
Arrest to TTM target	648.40(319.11)	659.3(368.3)	0.6689

Abbreviations: OHCA: out-of-hospital cardiac arrest; IHCA: in-hospital cardiac arrest; APACHE II: acute physiology and chronic health evaluation II; BT: body temperature; ROSC: return of spontaneous circulation; CPR: cardiopulmonary resuscitation; TTM: targeted temperature management

Supplement S4. ROC curve analysis for optimal cut-off values of pre-induction and induction time.

(A) Time from ROSC to initial cooling (pre-induction time) – 28-day survival

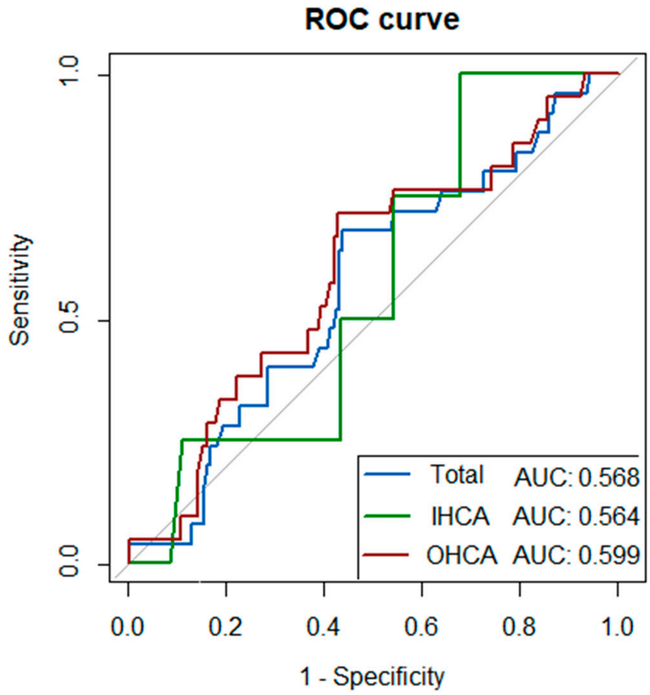

(B) Time from cooling to target temperature (induction time) – 28-day survival

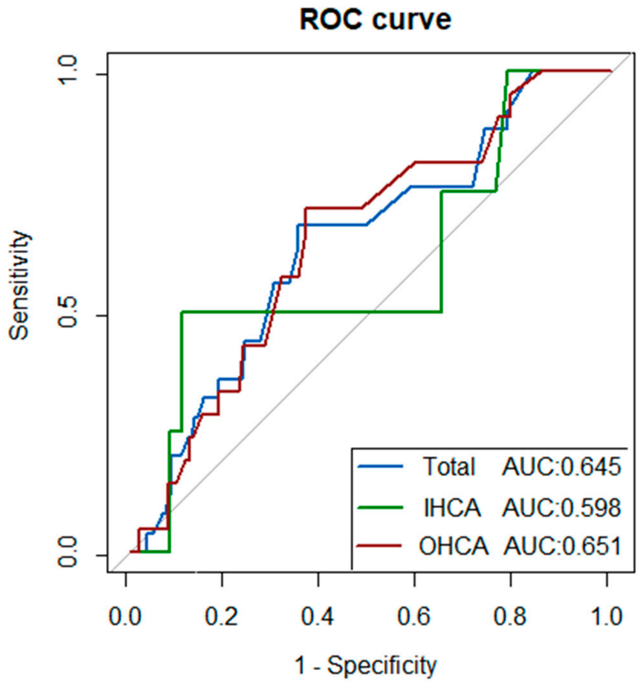

Abbreviations: OHCA: out-of-hospital cardiac arrest; IHCA: in-hospital cardiac arrest; ROSC: return of spontaneous circulation; ROC: receiver operating characteristic

Supplement S5. Kaplan–Meier curves of the cumulative probability of survival to day 90 (A, B) and day 180 (C, D) after cardiac arrest according to different pre-induction (A, C) and induction time (B, D).

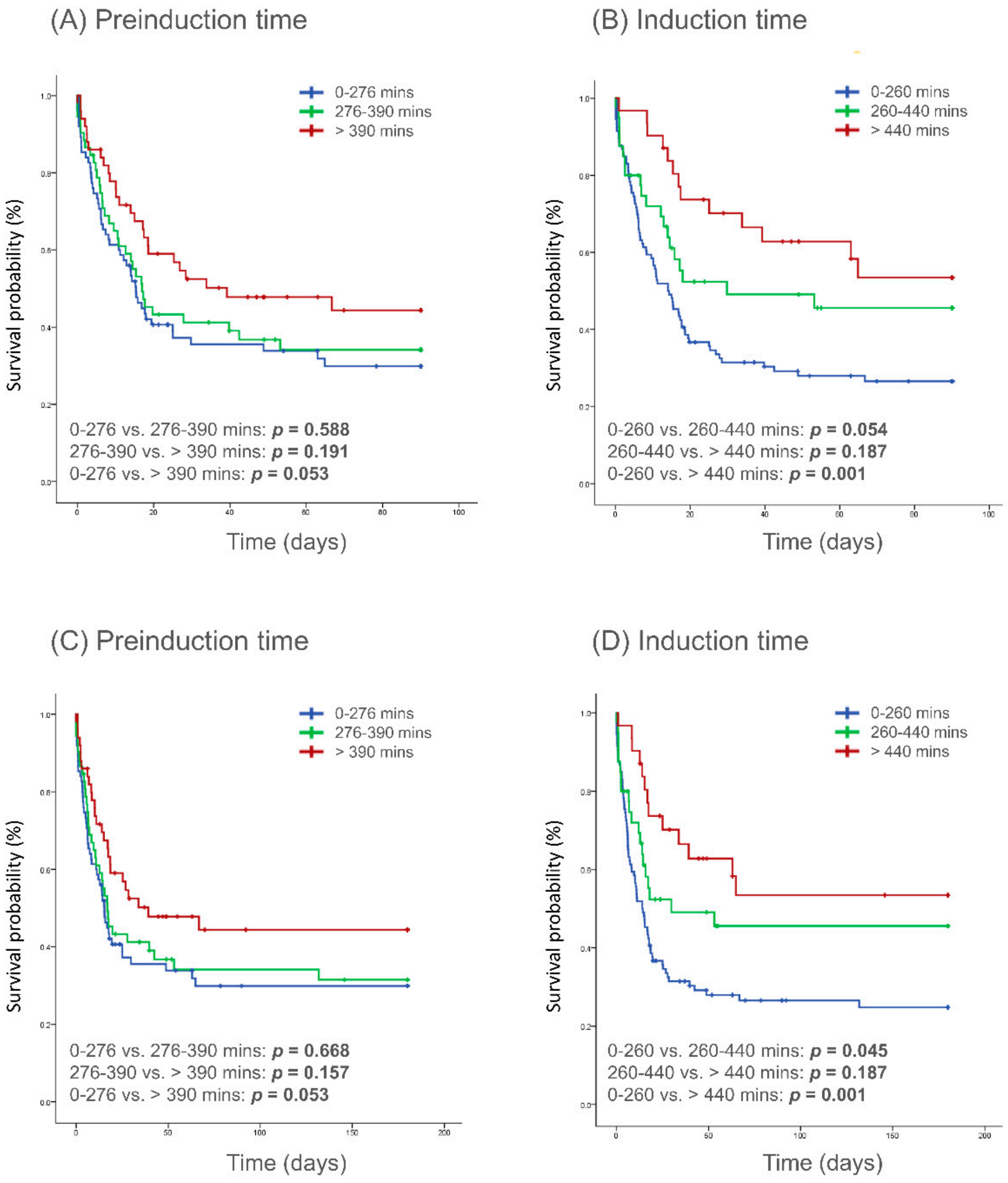

Supplement: Supplementary file 1 [file jcm-12-02628-s001.zip › jcm-2248616-supplementary.pdf]
